# LncRNA CASC2 inhibits hypoxia-induced pulmonary artery smooth muscle cell proliferation and migration by regulating the miR-222/ING5 axis

**DOI:** 10.1186/s11658-020-00215-y

**Published:** 2020-03-17

**Authors:** Yan Han, Yuhao Liu, Chaokuan Yang, Chuanyu Gao, Xiaoyan Guo, Jiangtao Cheng

**Affiliations:** Department of Cardiology, Henan Province People’s Hospital, Huazhongfuwai Hospital, No. 7, Weiwu Road, Jinshui area, Zhengzhou City, Henan P.R. China

**Keywords:** PAH, CASC2, miR-222, ING5, Proliferation, Migration

## Abstract

**Background:**

Pulmonary arterial hypertension (PAH) is often characterized by cell proliferation and migration of pulmonary arterial smooth muscle cells (PASMCs). LncRNA cancer susceptibility candidate 2 (CASC2) has been revealed to be involved in PASMC injury in hypoxia-induced pulmonary hypertension. However, the exact molecular mechanisms whereby CASC2 regulates PASMC proliferation and migration are still incompletely understood.

**Methods:**

The expression levels of CASC2, miR-222 and inhibitor of growth 5 (ING5) were measured using quantitative real-time polymerase chain reaction (qRT-PCR) or western blot, respectively. Cell proliferation was analyzed by Cell Counting Kit-8 (CCK-8) assay. Wound healing assay was used to analyze cell migration ability. The relationship between miR-222 and CASC2 or ING5 was confirmed using bioinformatics analysis, luciferase reporter assay and RNA immunoprecipitation assay.

**Results:**

CASC2 was down-regulated in hypoxia-induced PASMCs in a dose- and time-dependent manner. Functional experiments showed that CASC2 overexpression could reverse hypoxia-induced proliferation and migration of PASMCs. Bioinformatics analysis indicated that CASC2 acted as a competing endogenous RNA of miR-222, thereby regulating the expression of ING5, the downstream target of miR-222, in PASMCs. In addition, rescue assay suggested that the inhibition mediated by CASC2 of hypoxia-induced PASMC proliferation and migration could be attenuated by miR-222 inhibition or ING5 overexpression.

**Conclusion:**

CASC2 attenuated hypoxia-induced PASMC proliferation and migration by regulating the miR-222/ING5 axis to prevent vascular remodeling and the development of PAH, providing a novel insight and therapeutic strategy for hypoxia-induced PAH.

## Background

Pulmonary arterial hypertension (PAH) is an irreversible life-threatening disease, characterized by pulmonary vasoconstriction and vascular remodeling, resulting in a progressive increase of pulmonary vascular resistance and pulmonary arterial pressure followed by right ventricular hypertrophy and ultimately heart failure and death [1]. The pathobiology of PAH is complex; excessive proliferation and migration of pulmonary arterial smooth muscle cells (PASMCs) induced by hypoxia can result in the narrowing or occlusion of pulmonary vessels, which are the main cellular mechanisms of pulmonary vascular remodeling and affect the initiation and progression of PAH [2, 3].

Long non-coding RNAs (lncRNAs) are non-coding, endogenous cellular RNAs longer than 200 nucleotides in length [4]. Previous research confirmed that lncRNAs participated in diverse biological processes, including cell-cycle control, epigenetic regulation, chromatin remodeling and differentiation [5, 6]. Additionally, abnormally expressed lncRNAs serve as tumor inhibitors or oncogenes involved in the pathogenesis of numerous diseases, including cancers and cardiovascular diseases [7, 8]. Recently, emerging evidence indicated that lncRNAs were associated with vascular pathophysiology and involved in regulating the behaviors of endothelial cells (ECs) and vascular smooth muscle cells (VSMCs), affecting the contractility of SMCs as well as the proliferation, apoptosis and inflammatory responses to VSMCs [4, 9, 10]. In addition, it has been indicated that lncRNA cancer susceptibility candidate 2 (CASC2) functioned as a cancer suppressor in human cancers [11]. For instance, CASC2 alleviated the growth and metastasis of oral squamous cell carcinoma via down-regulating cyclin-dependent kinase 1 (CDK1), a key player in cell cycle regulation [12]. Overexpression of CASC2 inhibited the progression of hepatocellular carcinoma cells [13]. CASC2 repressed cell proliferation, invasion and angiogenesis in cervical cancer by activating the MAPK pathway [14]. Gong et al. revealed that CASC2 could suppress PASMC proliferation and the phenotypic switch in hypoxia-induced pulmonary hypertension (PH), suggesting the possible regulatory role of CASC2 in the pathogenesis of PAH [15]. However, the exact molecular mechanisms of CASC2 in the development of PAH remain unclear.

Endothelium damage and vascular lumen stenosis often initiated and promoted hypertension, atherosclerosis, PAH and other cardiovascular diseases. Up to now, various microRNAs (miRNAs) have been implicated in the pathogenesis of these processes [9, 16]. MiR-222, a member of miRNAs, has been found to be involved in the development of multiple cancers [17, 18]. Additionally, accumulating studies have identified the involvement of miR-222 in vascular injury and remodeling. For example, miR-222 inhibited proliferation of vascular ECs, and promoted proliferation of VSMCs [19, 20]. Inhibitor of growth 5 (ING5) is a member of the ING candidate tumor suppressor family, participating in the control of multiple cellular functions, such as the modulation of cell growth, apoptosis, differentiation, the cell cycle, DNA damage repair, and chromatin remodeling [21]. Recently, Zhu et al. found that ING5 was a target of lncRNA urothelial carcinoma associated 1 (UCA1), an important regulator in the tumorigenesis of many cancers, and might be involved in the UCA1-mediated promotion of the proliferation of hypoxic human PASMCs [22].

In the present study, we focused on the expression pattern of CASC2 in hypoxia-induced PASMCs, and explored the regulatory role as well as the underlying molecular mechanisms of CASC2 in hypoxia-induced vascular remodeling.

## Materials and methods

### Cell culture

Human PASMCs were purchased from American Type Culture Collection (ATCC; Rockville, MD, USA) and cultured in Dulbecco’s Modified Eagle’s Medium (DMEM; Gibco, Carlsbad, CA, USA) containing 10% fetal bovine serum (FBS; Gibco), 1% streptomycin and 1% penicillin in a humidified 5% CO_2_ at 37 °C.

### Human serum collection

Serum samples were obtained from healthy participants (*n* = 10) and patients with PAH (*n* = 30) at Henan Province People’s Hospital, Huazhongfuwai Hospital in line with the guidelines approved by the Ethics Committee of Henan Province People’s Hospital, Huazhongfuwai Hospital. All subjects had signed written informed consent. After standing at room temperature for 1 h, serum samples were centrifuged at 3000 g for 10 min at 4 °C and immediately saved at − 80 °C.

### Hypoxia treatment

For the hypoxic stimulation, PASMCs were cultured in a hypoxic incubator which was constantly infused with 85% N_2_ + 5% CO_2_ + 10% O_2_, 92% N_2_ + 5% CO_2_ + 3% O_2_ or 94% + N_2_ + 5% CO_2_ + 1% O_2_ for 48 h at 37 °C. For normoxia experiments, PASMCs were incubated with constantly infused air (21% O_2_ + 5% CO_2_ + 74% N_2_) at 37 °C.

### Cells transfection

MiR-222 mimic (miR-222), mimic negative control (miR-NC), miR-222 inhibitor (anti-miR-222) and inhibitor negative control (anti-miR-NC) were purchased from RIBOBIO (Guangzhou, China). The pcDNA3.1-CASC2 overexpression vector (CASC2), pcDNA3.1-ING5 overexpression vector (ING5), pcDNA3.1 empty vector (pcDNA), small interfering RNA (siRNA) against CASC2 (si-CASC2) and siRNA negative control (si-NC) were obtained from Genepharma (Shanghai, China). All the oligonucleotides or vectors were transfected using Lipofectamine 3000 (Invitrogen, Carlsbad, CA, USA) according to the instructions of the manufacturer.

### Quantitative real-time polymerase chain reaction (qRT-PCR)

Total RNA from PASMCs was isolated using Trizol reagent (Invitrogen) following the standard protocol. For detecting the expression of CASC2 and ING5, PrimeScript RT reagent kit (Takara, Dalian, China) was used to synthesize cDNA. For detecting miR-222 expression, the high-capacity cDNA reverse transcription kit (Thermo Fisher Scientific, Waltham, MA, USA) was used for cDNA synthesis. Subsequently, quantitative PCR was performed using the SYBR Green PCR Master Mix kit (Takara). The fold change was normalized using GAPDH or U6 and quantified by the 2^-ΔΔCt^ method. The primers were as follows: CASC2, F 5′-TACAGGACAGTCAGTGGTGGTA-3′, R 5′-ACATCTAGCTTAGGAATGTGGC-3′; ING5, F 5′-TCCAGAACGCCTACAGCAAG-3′, R 5′-TGCCCTCCATCTTGTCCTTC-3′; GAPDH, F 5′-AGTGGCAAAGTGGAGATT-3′, R 5′-GTGGAGTCATACTGGAACA-3′; miR-222, F 5′-CCCTCAGTGGCTCAGTAG-3′, R 5′-CCACCAGAGACCCAGTAG-3′; U6, F 5′-CTCGCTTCGGCAGCACA-3′, R 5′-AACGCTTCACGAATTTGCGT-3′.

### Cell proliferation assay

Following the transfection or treatment, viability of PASMCs was examined using Cell Counting Kit-8 (CCK-8) assay. PASMCs (5000 cells per well) were plated in 96-well plates overnight, and then was incubated with 10 μL of CCK-8 solution (Thermo Fisher Scientific) per well at 37 °C for 4 h. Finally, the absorbance at 450 nm in each well was detected by a spectrophotometer (Bio-Rad, Hercules, CA, USA).

### Wound healing assay

Migration ability of PASMCs was determined by wound healing assay. PASMCs were cultured on 6-well plates. Then a vertical line was scratched using a pipette in the cell plate. After removing cell debris using PBS, cells were cultured in fresh serum-free medium for 24 h. Finally, the wounded areas were observed and photographed and the migratory distance of PASMCs was measured to quantify the migration rate of the cells.

### RNA immunoprecipitation (RIP)

RIP assay was conducted using a Magna RNA immunoprecipitation kit (Millipore, Billerica, MA, USA). PASMCs were lysed with RIP buffer and then incubated with magnetic beads coated with anti-Ago2 or IgG antibody. Finally, the immunoprecipitated RNAs were isolated using TRIzol reagent and the enrichment was analyzed with qRT-PCR.

### Luciferase reporter assay

The CASC2 mRNA and ING5 3′-UTR containing wild-type (WT) or their mutant (MUT) binding sequence of miR-222 were cloned into the luciferase reporter construct pmiR-RB-Report (Promega, Shanghai, China). After that, PASMCs were co-transfected with miR-222 mimic or miR-NC mimic and corresponding luciferase reporters, or a control luciferase plasmid using Lipofectamine 3000. Following 48 h incubation, a dual luciferase assay kit (Promega) was used to analyze the luciferase activity in accordance with the protocols of the manufacturer.

### Western blot assay

Western blot assays were performed in strict accordance with standard steps. Western blot assay was performed using primary antibodies against ING5 (Abcam, 1:800), β-actin (Abcam, 1:200) as well as HRP-conjugated secondary antibody (Abcam, 1:2000). The protein signal was visualized by chemiluminescence chromogenic substrate (Beyotime, Shanghai, China).

### Statistical analysis

Statistical analysis was implemented using GraphPad Prism 7 (GraphPad Inc., San Diego, CA, USA) and all data from at least three independent experiments were presented as the mean ± standard deviation (SD). The correlation analysis was analyzed using Spearman’s correlation test. Student’s *t*-test and two-way analysis of variance (ANOVA) followed by Dunnett’s test or two-tailed t-tests were used to analyze the significance of differences between groups. *P* < 0.05 indicated a statistically significant difference.

## Results

### CASC2 is down-regulated in hypoxia-induced PASMCs

**Fig. 1:**
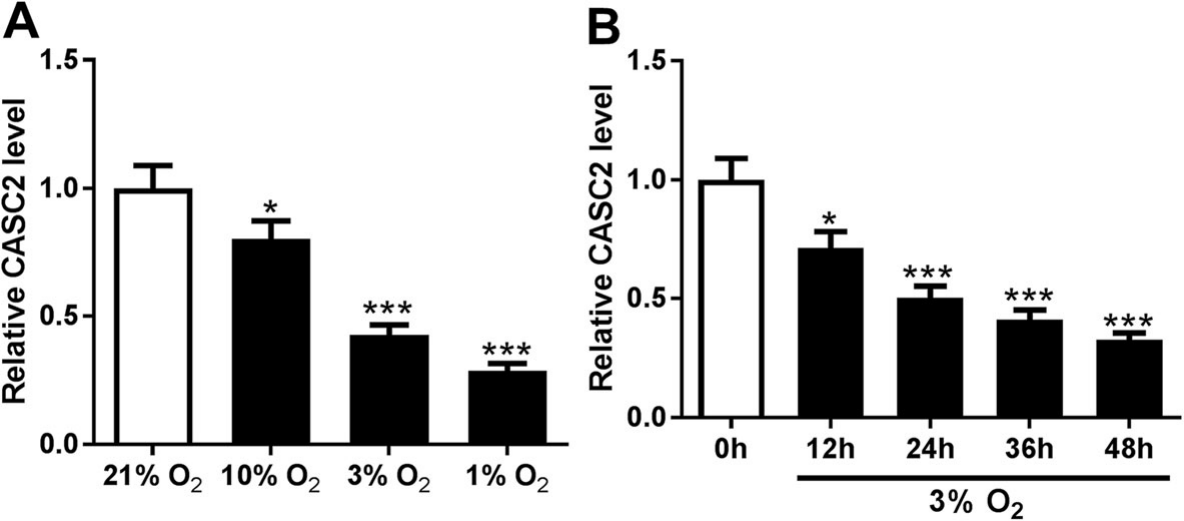
CASC2 is down-regulated in hypoxia-induced PASMCs. **a** Expression of CASC2 was detected using qRT-PCR in PASMCs following O_2_ exposure (21, 10, 3 and 1%) for 24 h. **b** CASC2 expression in PASMCs exposed to 3% O_2_ for 0 h, 12 h, 24 h, 36 h and 48 h was measured by qRT-PCR. Experiments were performed in triplicate. *P < 0.05, ***P < 0.001

The expression of CASC2 in PASMCs under normoxia or hypoxia conditions was detected by qRT-PCR and the results showed that CASC2 level was significantly decreased in PASMCs exposed to hypoxia for 24 h in a dose-dependent manner compared with that in the normoxic control (21% O_2_) (Fig. 1a). In addition, the expression of CASC2 was also time-dependently down-regulated in PASMCs by 3% hypoxia exposure at 0 h, 12 h, 24 h, 36 h and 48 h (Fig. 1b). Thus, these findings verified that CASC2 was down-regulated in hypoxia-induced PASMCs and aberrantly expressed CASC2 might be related to the pathogenesis of PAH.

### Downregulated CASC2 promotes proliferation and migration of hypoxia-induced PASMCs

**Fig. 2:**
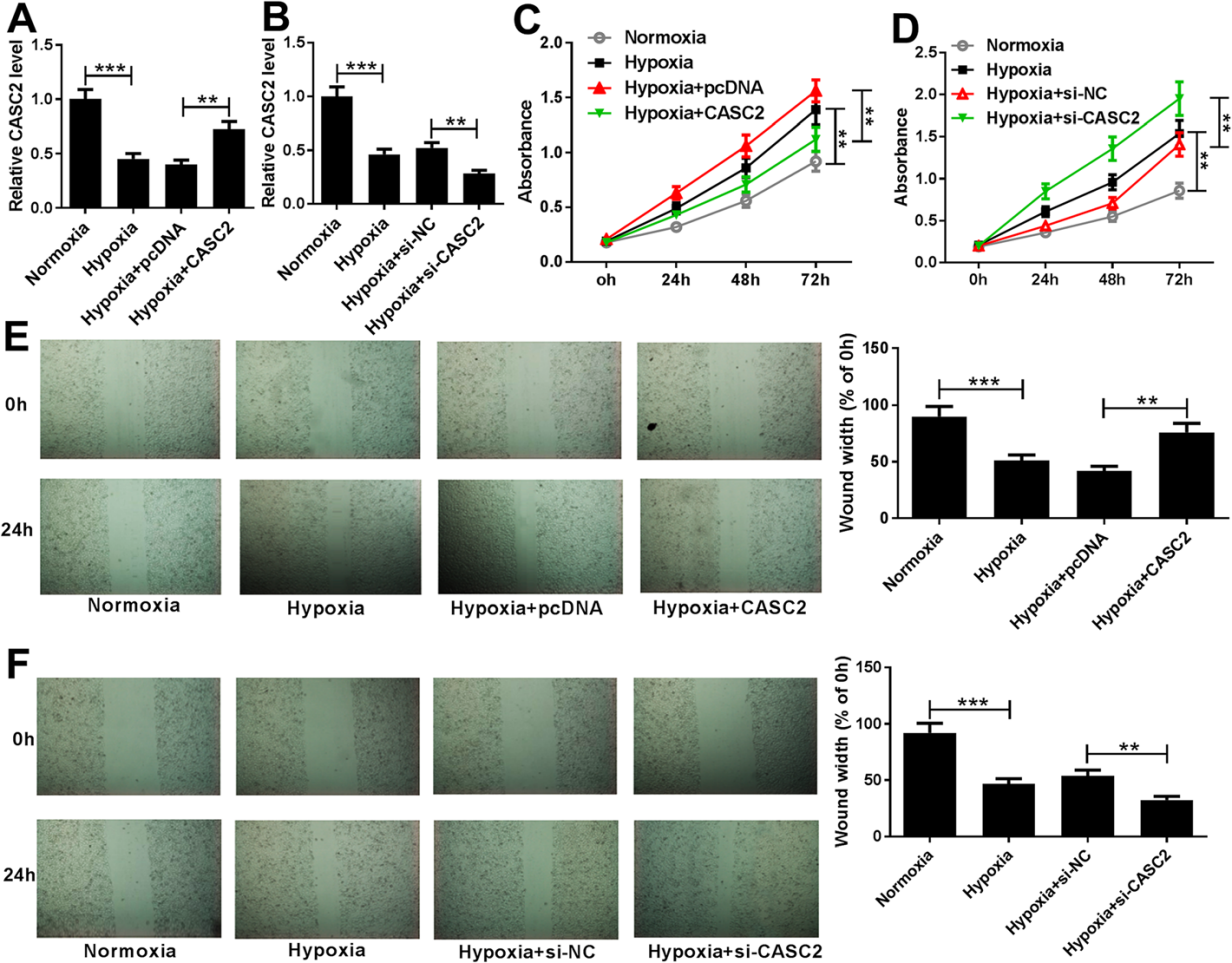
Overexpressed CASC2 suppresses hypoxia-induced PASMC proliferation and migration. PASMCs were transfected with CASC2 or si-CASC2 prior to exposure to hypoxia (3% O_2_) or normoxia for 24 h in triplicate. **a**, **b** Expression of CASC2 was detected by qRT-PCR in hypoxia- or normoxia-induced PASMCs. **c**, **d** Proliferation was determined using CCK-8 assay, and data were derived from an average of three independent experiments. **e**, **f** Wound healing assay was used to detect hypoxia-induced PASMC migration, and experiments were performed in triplicate. **P < 0.01, ***P < 0.001

To explore the potential biological functions of CASC2 in proliferation and migration of hypoxia-induced PASMCs, PASMCs were transfected with CASC2 or si-CASC2 prior to exposure to the hypoxia condition (3% O_2_), and then the transfection efficiency was determined using qRT-PCR with the results of decreased CASC2 expression in PASMCs transfected with si-CASC2 and increased CASC2 expression in PASMCs transfected with CASC2 (Fig. 2a, b). Afterwards, CCK-8 assay demonstrated that hypoxia induced PASMCs proliferation, while this promotion was alleviated by overexpressed CASC2, and aggravated by decreased CASC2 (Fig. 2c, d). Subsequently, wound healing assay showed that high CASC2 expression reversed hypoxia-induced migration of PASMCs, while low CASC2 expression showed opposite effects (Fig. 2e, f). In all, CASC2 could suppress hypoxia-induced PASMC proliferation and migration.

### CASC2 is a sponge of miR-222

**Fig. 3:**
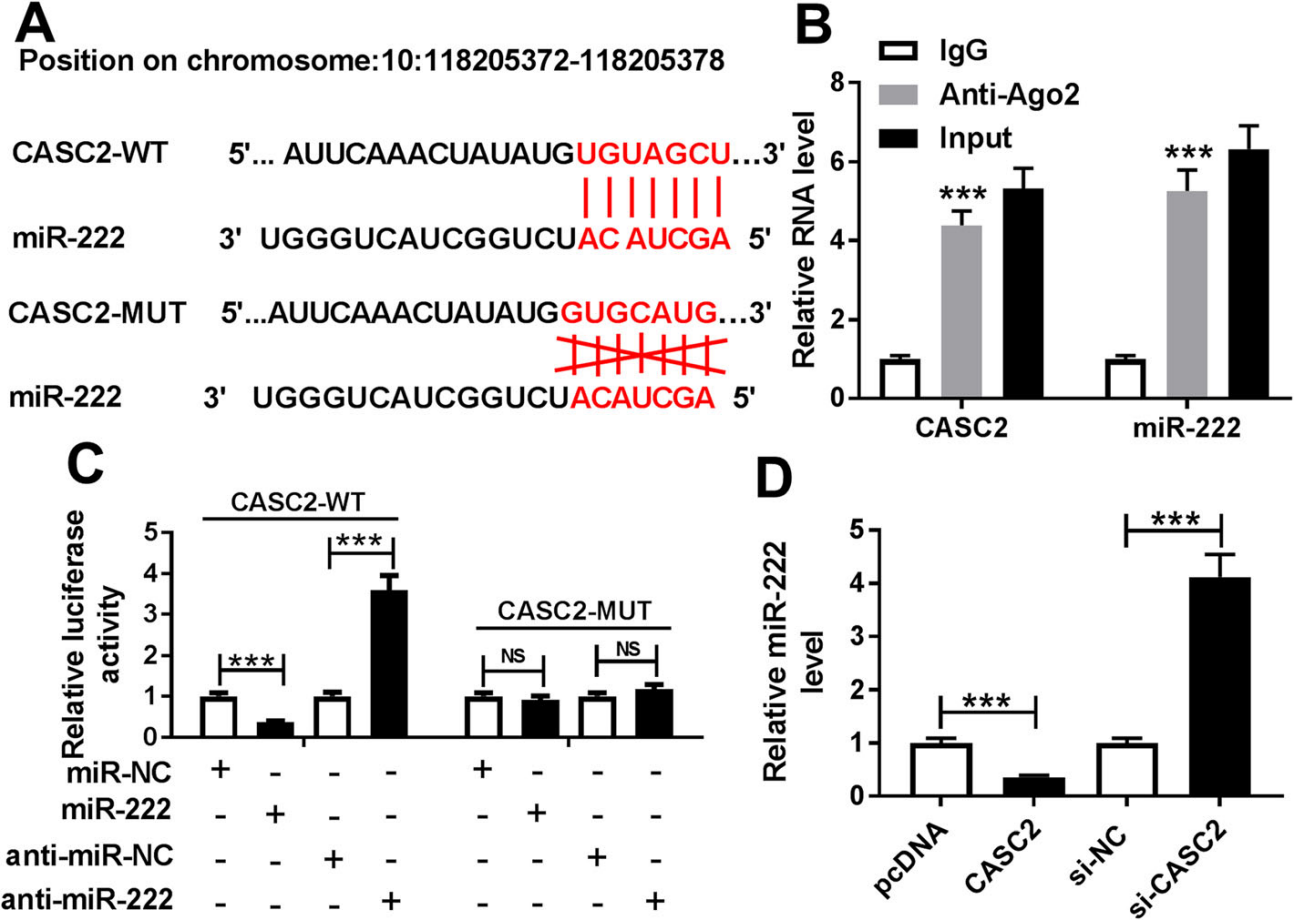
CASC2 is a sponge of miR-222. **a** Potential binding sites of miR-222 in CASC2 were predicted by bioinformatics analysis. **b** Enrichment of CASC2 and miR-222 was analyzed in PASMCs after Ago2 RIP. **c** Luciferase activity was measured in PASMCs co-transfected with CASC2-WT or CASC2-MUT and miR-NC, miR-222 or anti-miR-NC, anti-miR-222. **d** MiR-222 expression in PASMCs was examined after transfection with CASC2 or si-CASC2 using qRT-PCR, and results were presented as the average of three independent replicates. ***P < 0.001

To further investigate the underlying regulatory mechanism of CASC2-mediated PASMC proliferation and migration, the potential targets of CASC2 were predicted through the LncBase V.2 database, and CASC2 contained the binding sequences of miR-222 (Fig. 3a); thus we hypothesized that miR-222 might be a target of CASC2. Subsequently, RIP assay showed significant enrichment of CASC2 and miR-222 in PASMCs after Ago2 RIP, whereas its efficacy was lost in response to IgG RIP, indicating the direct interaction between miR-222 and CASC2 (Fig. 3b). Moreover, luciferase reporter assay showed that miR-222 mimic reduced the luciferase activity of the CASC2-WT reporter vector but not the CASC2-MUT reporter vector in PASMCs, while miR-222 inhibitor increased the luciferase activity of the CASC2-WT reporter vector but not the CASC2-MUT reporter in PASMCs (Fig. 3c), further suggesting that miR-222 was a target of CASC2 in PASMCs. Additionally, we discovered that the expression of miR-222 was inhibited by CASC2 up-regulation, but was enhanced by CASC2 down-regulation in PASMCs. All the evidence suggested that CASC2 directly bound to miR-222 and negatively regulated its expression.

### CASC2 suppresses hypoxia-induced proliferation and migration of PASMCs by regulating miR-222 expression

**Fig. 4:**
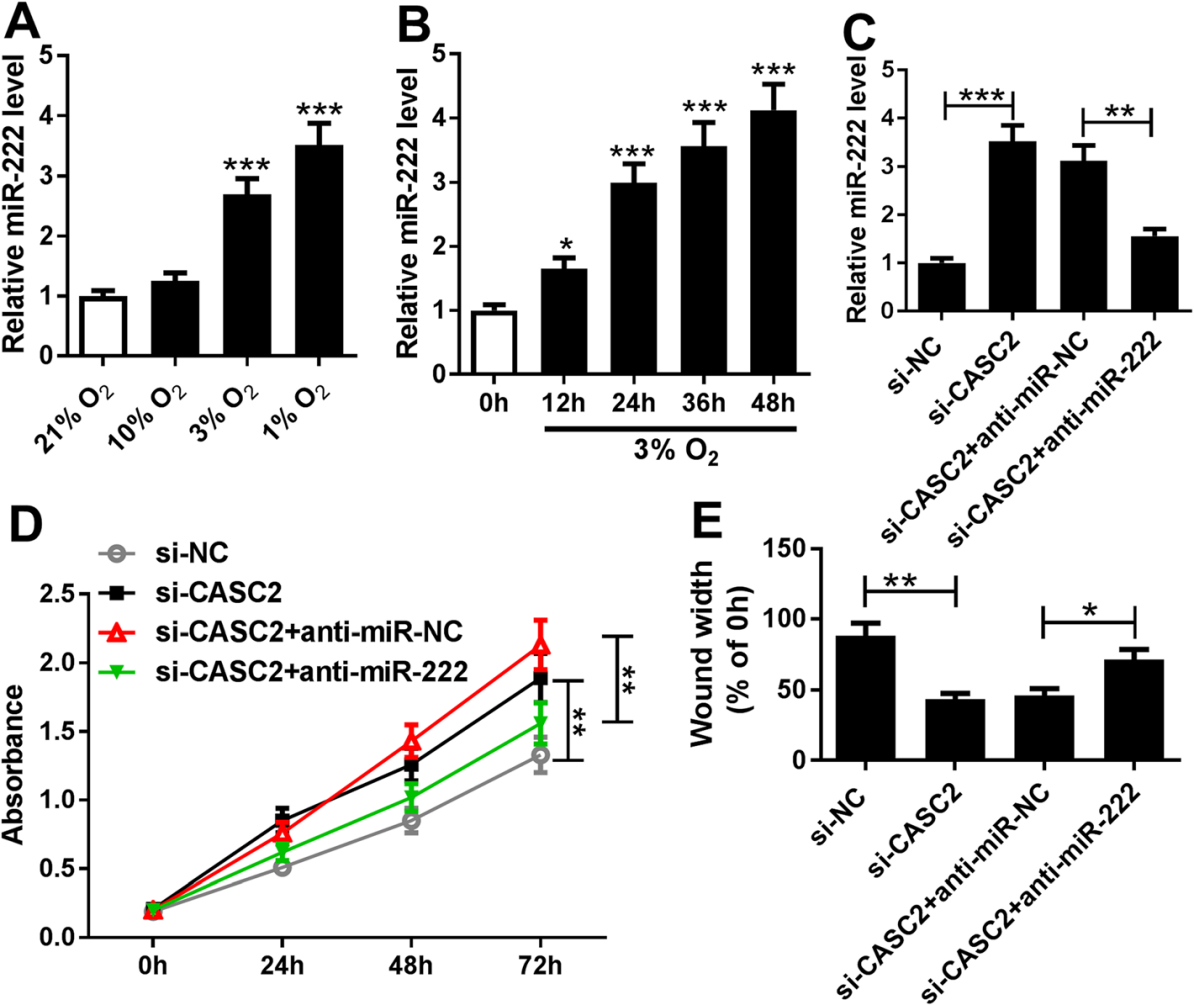
CASC2 suppresses hypoxia-induced PASMC proliferation and migration by regulating miR-222 expression. **a** Expression of miR-222 was detected using qRT-PCR in PASMCs following O_2_ exposure (21, 10, 3 and 1%) for 24 h. **b** MiR-222 expression in PASMCs exposed to 3% O_2_ for 0 h, 12 h, 24 h, 36 h and 48 h was measured by qRT-PCR. Experiments were performed in triplicate. **c** Expression of miR-222 was detected by qRT-PCR in hypoxia-induced PASMCs transfected with si-NC, si-CASC2, si-CASC2 + anti-miR-NC or si-CASC2 + anti-miR-222 three times. **d** CCK-8 assay was used to determine the proliferation of hypoxia-induced PASMCs; experiments were conducted at least three times. **e** Migration ability of hypoxia-induced PASMCs was measured using wound healing assay; bar charts represent relative migrated cells from three independent experiments. *P < 0.05, **P < 0.01, ***P < 0.001

We further elucidated whether miR-222 was involved in CASC2-mediated regulation of PASMCs. Firstly, the expression of miR-222 was measured in PASMCs under the normoxia or hypoxia condition, and the results demonstrated that hypoxia induced miR-222 expression in PASMCs in a dose- and time-dependent manner (Fig. 4a, b). Subsequently, the effects of miR-222 inhibition under hypoxia (3% O_2_) was investigated. As shown in Fig. S1 B and D, miR-222 inhibition reversed hypoxia-induced PASMC proliferation and migration, suggesting that miR-222 was an important regulator in hypoxia-induced PASMC injury. Then, the rescue experiment was performed. PASMCs exposed to 3% O_2_ were transfected with si-NC, si-CASC2, si-CASC2 + anti-miR-NC or si-CASC2 + anti-miR-222, and the transfection efficiency was determined using qRT-PCR through evaluating the miR-222 expression (Fig. 4c). Subsequently, CCK-8 assay and wound healing assay results showed that miR-222 inhibition could attenuate the CASC2 deletion-mediated promotion of the proliferation and migration of hypoxia-induced PASMCs (Fig. 4d, e). Thus, we concluded that CASC2 regulated hypoxia-induced PASMC proliferation and migration by binding to miR-222.

### ING5 is a target of miR-222

**Fig. 5:**
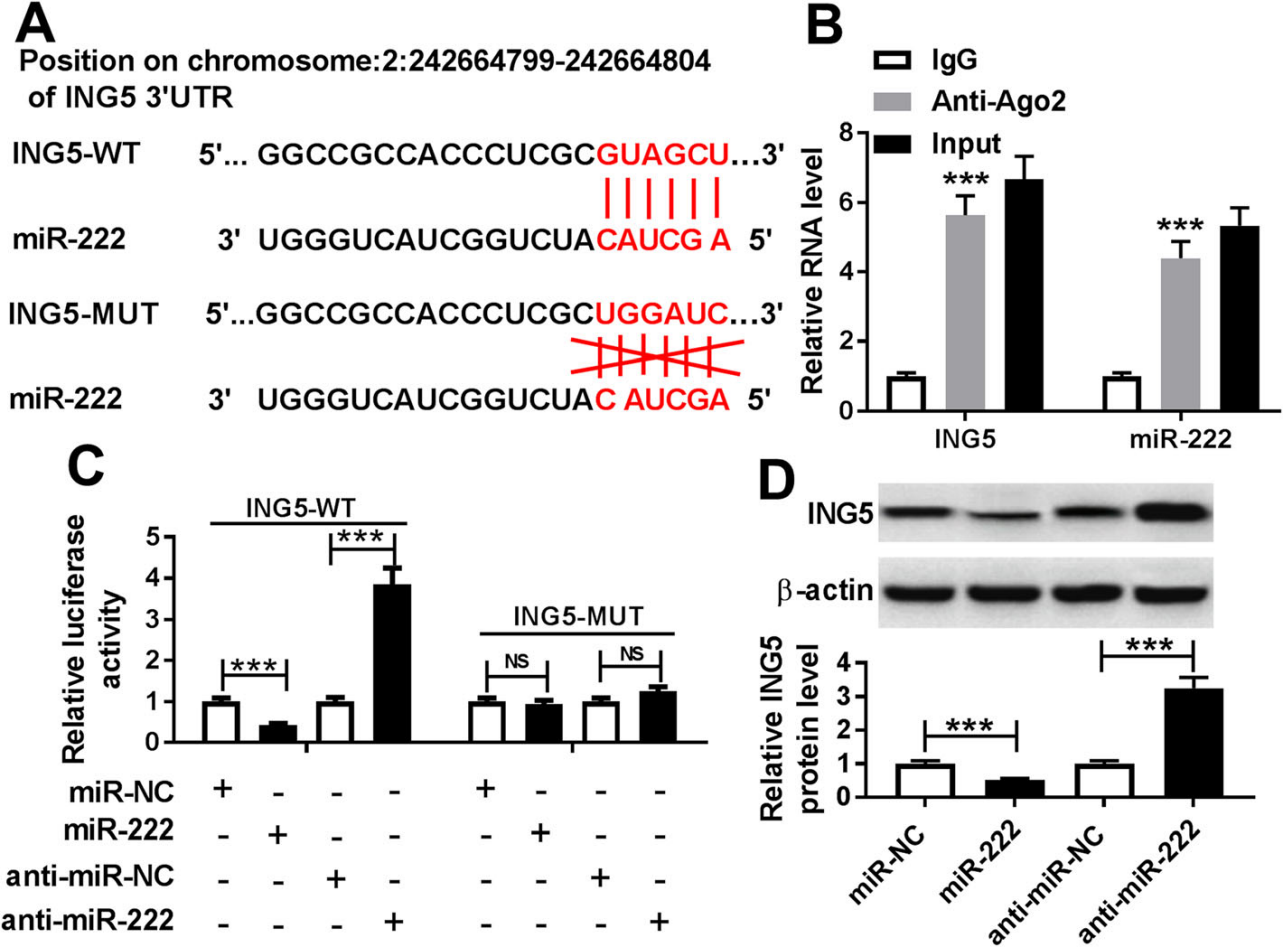
ING5 is a target of miR-222. **a** Potential binding sites between miR-222 and ING5 were exhibited. **b** Enrichment of ING5 and miR-222 was detected in PASMCs after Ago2 RIP. **c** Luciferase activity was analyzed in PASMCs co-transfected with ING5-WT or ING5-MUT and miR-NC, miR-222 or anti-miR-NC, anti-miR-222. **d** ING5 protein expression in PASMCs transfected with miR-222 or anti-miR-222 was examined three times by western blot assay. **P < 0.01, ***P < 0.001

According to the prediction of the microT-CDS database, ING5 contained binding sequences of miR-222 (Fig. 5a). Immediately, RIP assay confirmed the relationship between miR-222 and ING5 due to a significant enrichment of ING5 and miR-222 in PASMCs after Ago2 RIP (Fig. 5b). Also, luciferase reporter assay showed that the relative luciferase activity of ING5-WT reporter vector was obviously reduced by the miR-222 mimic, whereas it was enhanced by the miR-222 inhibitor in PASMCs, and no significant change was observed in ING5-MUT reporter in PASMCs co-transfected with miR-222 or anti-miR-222 (Fig. 5c), further suggesting the interaction between miR-222 and ING5. Meanwhile, we found that overexpressed miR-222 inhibited ING5 protein expression, while down-regulated miR-222 stimulated ING5 protein expression in PASMCs (Fig. 5d). These findings indicated that miR-222 targetedly suppressed ING5 expression in PASMCs.

### MiR-222 promotes hypoxia-induced PASMC proliferation and migration by targeting ING5

**Fig. 6:**
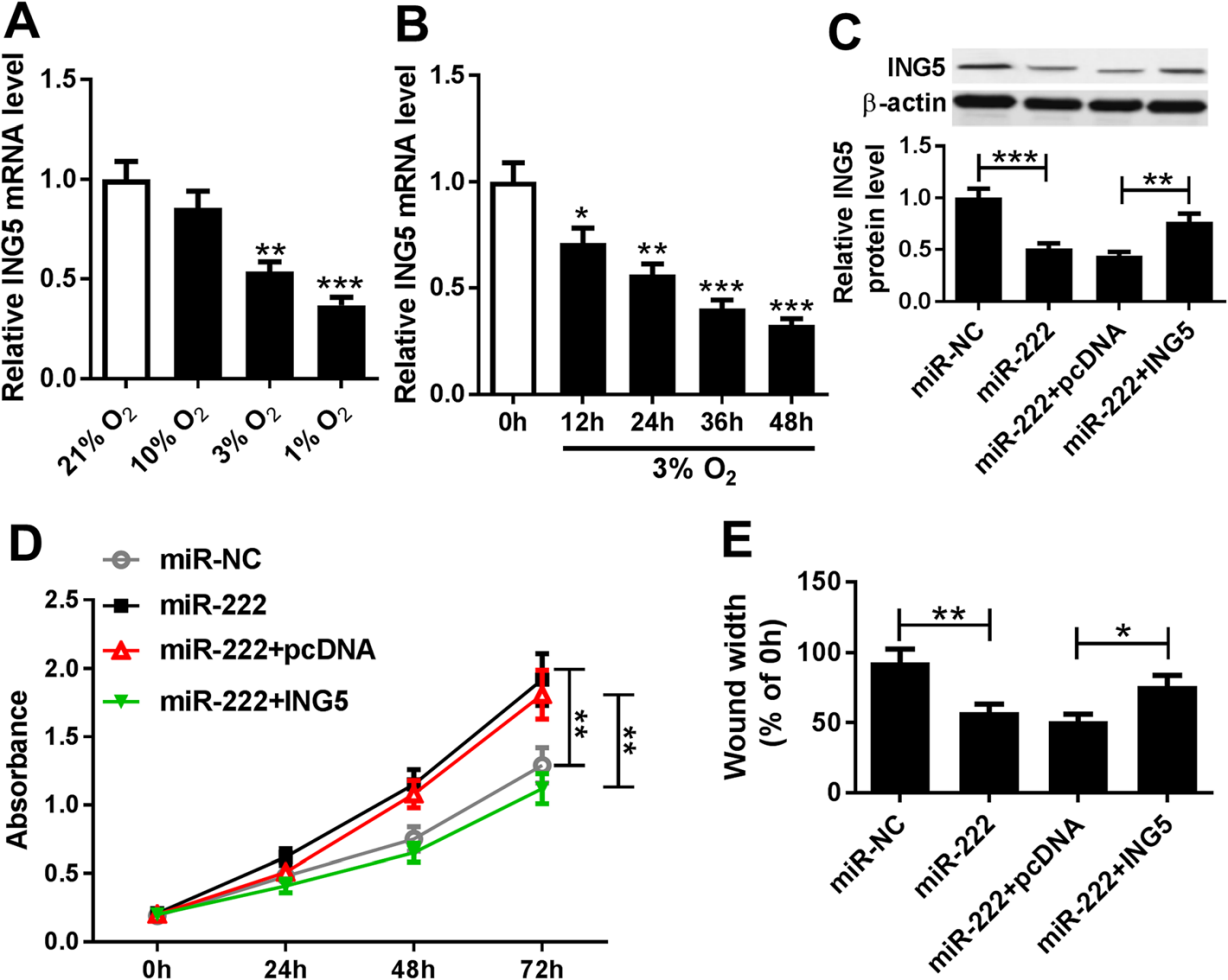
MiR-222 promotes hypoxia-induced PASMC proliferation and migration by regulating ING5 expression. **a** mRNA expression of ING5 was detected using qRT-PCR in PASMCs following O_2_ exposure (21, 10, 3 and 1%) for 24 h. **b** ING5 mRNA expression in PASMCs exposed to 3% O_2_ for 0 h, 12 h, 24 h, 36 h and 48 h was measured by qRT-PCR. **c** Protein expression of ING5 was determined by western blot assay in hypoxia-induced PASMCs after transfection with miR-NC, miR-222, miR-222 + pcDNA or miR-222 + ING5, protein bands were quantified and a bar chart showing relative protein levels was derived from triplicate samples. **d** CCK-8 assay was performed to determine the proliferation of hypoxia-induced PASMCs after transfection; data are derived from three independent experiments. **e** Migration ability of hypoxia-induced PASMCs was analyzed using wound healing assay, and results were presented as the average of three independent replicates. *P < 0.05, **P < 0.01, ***P < 0.001

The expression of ING5 was examined in PASMCs in the normoxia or hypoxia condition, and ING5 was found to be down-regulated in hypoxia-induced PASMCs in a dose- and time-dependent manner (Fig. 6a, b). Moreover, as presented in Fig. S1A and C, ING5 overexpression reversed hypoxia-induced PASMC proliferation and migration. All these results indicated that ING5 participated in hypoxia-induced PASMC injury. Subsequently, miR-NC, miR-222, miR-222 + pcDNA or miR-222 + ING5 was transfected into PASMCs before exposure to 3% O_2_, then the expression of ING5 protein was measured using western blot assay to determine the transfection efficiency (Fig. 6c). Moreover, the proliferation and migration abilities of hypoxia-induced PASMCs were investigated and we discovered that overexpressed miR-222 promoted hypoxia-induced PASMC proliferation and migration, which could be abated by highly expressed ING5 (Fig. 6d, e). Therefore, we revealed that miR-222 could promote hypoxia-induced PASMC proliferation and migration by regulating ING5 expression.

### CASC2 can regulate ING5 expression by directly binding to miR-222

**Fig. 7:**
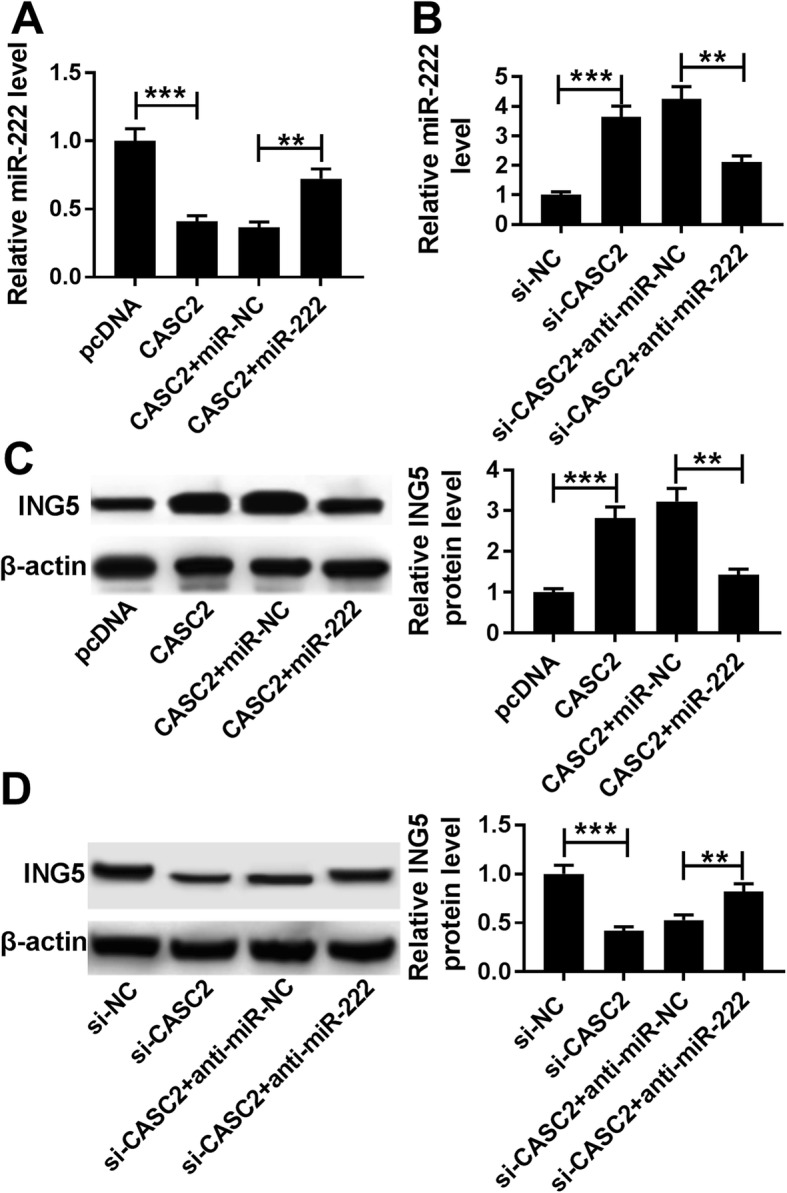
CASC2 can regulate ING5 expression by directly binding to miR-222. **a**, **c** Expression of miR-222 and ING5 protein in PASMCs transfected with pcDNA, CASC2, CASC2 + miR-NC or CASC2 + miR-222 was detected using qRT-PCR or western blot, respectively. **b**, **d** Levels of miR-222 and ING5 protein in PASMCs transfected with si-NC, si-CASC2, si-CASC2 + anti-miR-NC or si-CASC2 + anti-miR-222 were examined by qRT-PCR or western blot, respectively. The same experiment was repeated three times, and the average was taken. **P < 0.01, ***P < 0.001

Based on the above results, we investigated the regulatory relationship among CASC2, miR-222 and ING5 in PASMCs. The results showed that overexpressed CASC2 inhibited miR-222 expression, which could be restored by the miR-222 mimic (Fig. 7a). Meanwhile, down-regulated CASC2 promoted miR-222 expression, which could be reversed by the miR-222 inhibitor (Fig. 7b). In addition, we observed that the protein expression of ING5 was enhanced by CASC2 up-regulation and reduced by CASC2 downregulation, while these effects could be attenuated by up- or down-regulated miR-222, respectively (Fig. 7c, d). Taken together, CASC2 regulated ING5 expression by serving as a sponge of miR-222 in PASMCs.

### CASC2 attenuates hypoxia-induced PASMC proliferation and migration by regulating ING5 expression

**Fig. 8:**
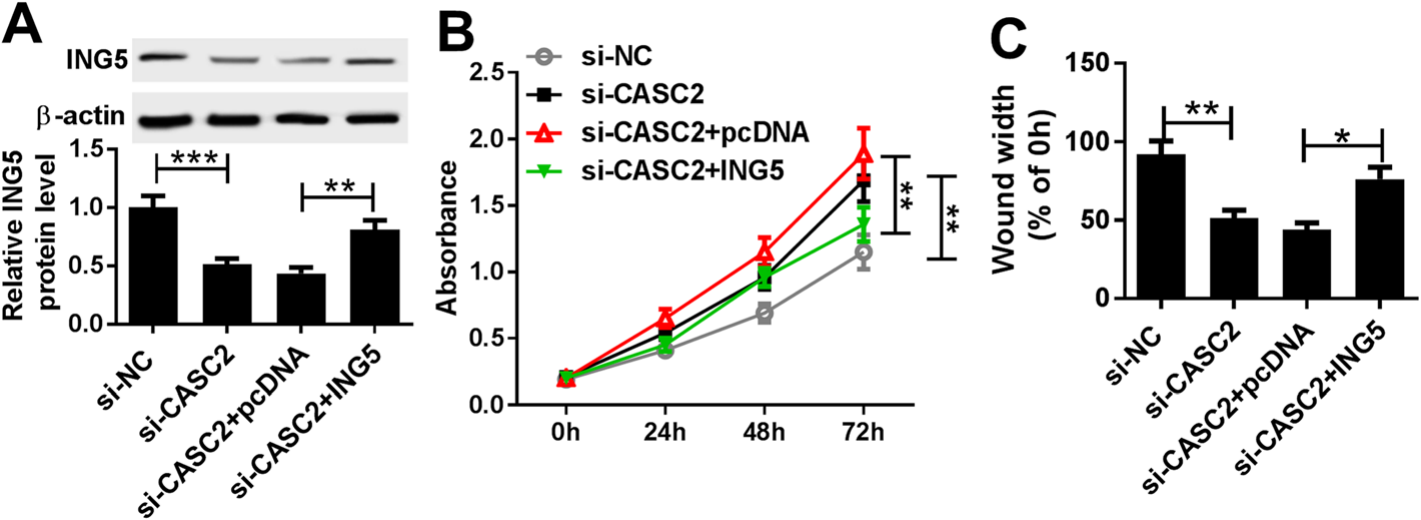
CASC2 can exert its inhibitory effect on proliferation and migration of hypoxia-induced PASMCs by regulating ING5 expression. PASMCs were transfected with si-NC, si-CASC2, si-CASC2 + pcDNA or si-CASC2 + ING5 in triplicate. **a** Protein expression of ING5 was measured by western blot in hypoxia-induced PASMCs after transfection, and the average from three independent experiments was taken. **b** CCK-8 assay was carried out to determine the proliferation of hypoxia-induced PASMCs after transfection, and all experiments were repeated three times independently. **c** Migration ability of hypoxia-induced PASMCs after transfection was analyzed using wound healing assay, and data are derived from an average of three independent experiments. **P < 0.01

To investigate whether ING5 was involved in CASC2-mediated inhibitory effects on hypoxia-induced PASMC impairment, PASMCs were transfected with si-NC, si-CASC2, si-CASC2 + pcDNA or si-CASC2 + ING5 before treatment with 3% O_2._ After transfection, the protein expression of ING5 was detected to verify the transfection efficiency (Fig. 8a). Subsequently, the rescue experiment showed that CASC2 down-regulation promoted hypoxia-induced PASMC proliferation and migration, which were reversed by ING5 overexpression (Fig. 8b, c). Thus, we confirmed that CASC2 might inhibit hypoxia-induced PASMC proliferation and migration by regulating ING5 expression.

### Serum CASC2 may be a potential circulating biomarker for PAH

**Fig. 9:**
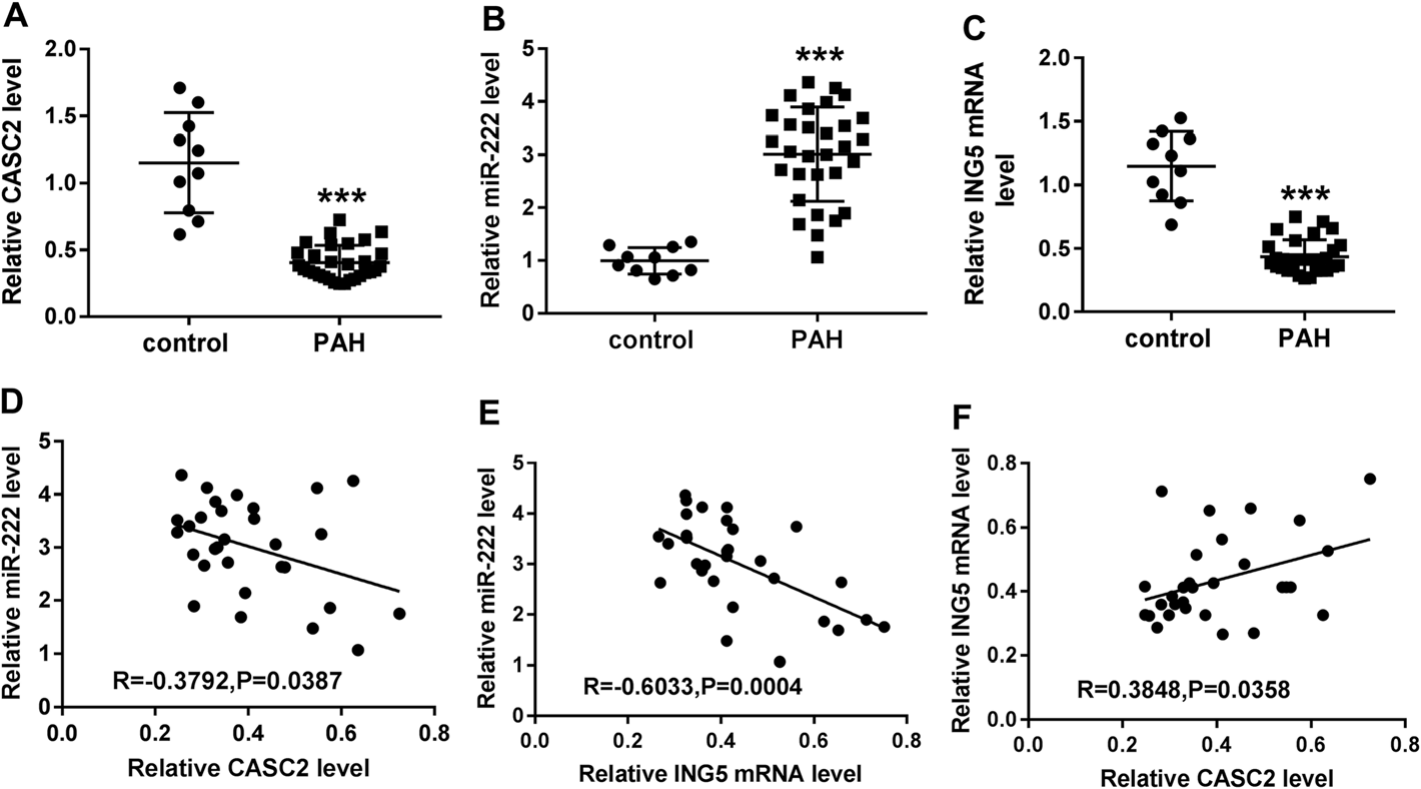
Expression and relationship of CASC2, miR-222 and ING5 in serum samples of PAH patients. **a-c** CASC2, miR-222 and ING5 expression levels were measured in the serum samples of PAH patients and were compared to the expression in healthy controls. **d-e** Correlation between CASC2 and miR-222 expression, miR-222 and ING5 expression, or ING5 and CASC2 expression in serum samples of PAH patients was analyzed using Spearman’s correlation test. Results are presented as the average of three independent replicates. ***P < 0.001

We further analyzed the level of these biomarkers in patients with PAH. The results showed that the expression of CASC2 and ING5 was lower (Fig. 9a, c), and miR-222 expression was higher (Fig. 9b) in the serum of PAH patients compared with the healthy controls. Also, a negative correlation between miR-222 and CASC2 (Fig. 9d) or ING5 (Fig. 9e) expression, and a positive correlation between CASC2 and ING5 expression (Fig. 9f) were observed in the serum of PAH patients. These data were of great clinical significance, which highlighted that CASC2 might regulate hypoxia-induced PASMC proliferation and migration by regulating the miR-222/ING5 axis in PAH.

## Discussion

PAH is a fatal disease with the hallmark of sustained pulmonary vasoconstriction and progressive pulmonary vascular remodeling. Although PAH treatment has achieved advanced development in current years, the prognosis related to PAH remains worse than numerous cancers [23]. Current pharmacological treatments of PAH are mainly vasodilators, and offer significantly increased survival, but there is still no cure other than transplantation, which suggests the urgent need for new effective biomarkers and therapeutic strategies [24]. In recent years, accumulating evidence has indicated that lncRNAs might exert vital effects on vascular pathophysiology and are involved in various pathogenic pathways, such as those underlying cardiovascular diseases and PAH [25]. For instance, LncRNA Hoxaas3 contributed to the proliferation of hypoxia-induced PASMCs and modulated cell cycle distribution in PH [26]. LncRNA MEG3 down-regulation triggered proliferation and migration of human PASMCs through regulating the p53 signaling pathway [27]. LncRNA MALAT1 contributed to PAH susceptibility in Chinese people by regulating proliferation and migration of vascular ECs [28]. All the research indicated lncRNAs as potential candidates for therapeutic intervention in PAH. Recently, lncRNA CASC2 has been revealed to suppress proliferation and phenotypic switch of PASMCs in hypoxia-induced PH [15]. However, the exact regulatory mechanisms of CASC2 in proliferation and migration of PASMCs in hypoxia-induced PAH remain unclear.

Hypoxia is a well-recognized stimulus for the development of PAH. In the present study, we found that CASC2 expression was significantly down-regulated in response to hypoxia in PASMCs in a dose- and time-dependent manner, and increased CASC2 suppressed hypoxia-induced PASMC proliferation and migration, which ultimately affected vascular remodeling, even the development of PAH. However, transfection of CASC2 exerted no significant effect on cell proliferation under normoxia in PASMCs.

Previous studies have reported that manipulation of miRNAs could reduce the burden of pathological vascular remodeling, and miRNAs were essential regulators of differentiation, development, phenotypic transformation and contractile function of VSMCs [29]. Additionally, the lncRNA-miRNA-mRNA axis has been highlighted for its important role in development of cardiovascular diseases [30]. MiR-222 has been identified to participate in many cardiac physiological functions and its deregulation is implicated in many cardiovascular diseases [31]. Liu et al. found that miR-222 was up-regulated in vascular walls with neointimal lesion formation, and knockdown of miR-222 repressed VSMC proliferation in vitro and in vivo [20]. Moreover, Xu et al. confirmed that miR-222 partially promoted PASMC proliferation via targeting P27 and TIMP3 in PAH [32]. In this study, bioinformatics analysis showed that CASC2 was confirmed to be a sponge of miR-222 and could negatively regulate its expression in PASMCs. Subsequently, miR-222 was found to be up-regulated in hypoxia-induced PASMCs in a dose- and time-dependent manner, suggesting that miR-222 might be implicated in hypoxia-induced PASMC injury, which was consistent with the previous study. Subsequently, rescue assay results showed that miR-222 inhibitor attenuated the promotion effects on proliferation and migration of hypoxia-induced PASMCs which were mediated by decreased CASC2. Therefore, we clarified that CASC2 could suppress proliferation and migration of hypoxia-induced PASMCs by regulating miR-222 expression.

**Fig. 10:**
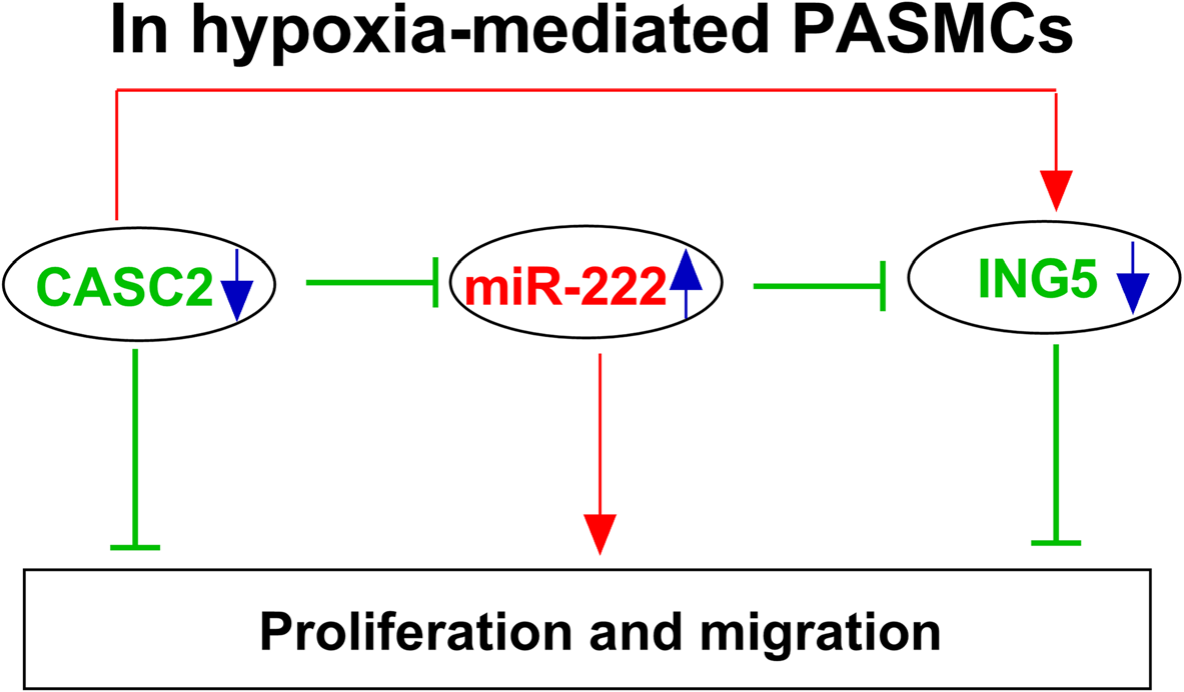
Schematic diagram of CASC2 regulation of proliferation and migration in hypoxia-induced PASMCs. CASC2 could regulate hypoxia-induced proliferation and migration of PASMCs by regulating miR-222/ING5 axis

ING5 often functioned as a tumor suppressor gene due to its inhibition of cell growth and promotion of cell apoptosis in various cancers [33, 34]. In a previous study, ING5 was found to serve as a target of UCA1 to restrain cell viability, but promote cell apoptosis in hypoxic human PASMCs, indicating the potential regulatory role of ING5 in hypoxia-induced PAH development [22]. In the current study, ING5 was also predicted and confirmed to be a target of miR-222 using bioinformatics analysis. Subsequently, the expression of ING5 was analyzed and the results showed that ING5 was down-regulated in hypoxia-induced PASMCs in a dose- and time-dependent manner. Subsequently, gain-of-function experiments were performed and the results demonstrated that miR-222 could promote hypoxia-induced proliferation and migration of PASMCs by regulating ING5 expression. Furthermore, we discovered that CASC2 acted as a competing endogenous RNA of miR-222, thereby regulating the expression of ING5 in PASMCs, indicating that ING5 might also be involved in the CASC2-mediated inhibitory effect on hypoxia-induced PASMCs. Additionally, we found that overexpressed ING5 could reverse CASC2 silence-mediated promotion of hypoxia-induced PASMC proliferation and migration. Thus, a CASC2/miR-222/ING5 axis in the regulation of hypoxia-induced PASMC injury was identified (Fig. 10).

## Conclusions

Our results demonstrated that CASC2 was down-regulated in response to hypoxia in PASMCs, and CASC2 could inhibit hypoxia-induced proliferation and migration of PASMCs by regulating the miR-222/ING5 axis to suppress vascular remodeling, indicating a novel insight and therapeutic strategy for hypoxia-induced PAH.

## Supplementary information

**Additional file 1 Figure S1.** Effects of miR-222 and ING5 on proliferation and migration of hypoxia-induced PASMCs. (A, C) Effects of miR-222 inhibition on proliferation and migration of normoxia- or hypoxia-induced PASMCs were detected using CCK-8 assay or wound healing assay. (B, D) Effects of ING5 overexpression on proliferation and migration of hypoxia-induced PASMCs were detected using CCK-8 assay or wound healing assay. Experiments were performed three times. *P < 0.05, **P < 0.01, ***P < 0.001.

## Data Availability

The data from this study are available in this published article.

## Abbreviations

CASC2: Cancer susceptibility candidate 2
CCK-8: Cell Counting Kit-8
CDK1: Cyclin-dependent kinase 1
ING5: Inhibitor of growth 5
PAH: Pulmonary arterial hypertension
PASMC: Proliferation and migration of pulmonary arterial smooth muscle cells
PASMCs: Pulmonary arterial smooth muscle cells
qRT-PCR: Quantitative real-time polymerase chain reaction
UCA1: Urothelial carcinoma associated 1
VSMCs: Vascular smooth muscle cells

## References

[1] Chen T, Huang JB, Dai J, Zhou Q, Raj JU, Zhou G (2018). PAI-1 is a novel component of the miR-17~ 92 signaling that regulates pulmonary artery smooth muscle cell phenotypes. Am J Phys Lung Cell Mol Phys.

[2] Nie Xiaowei, Chen Yuan, Tan Jianxin, Dai Youai, Mao Wenjun, Qin Guowei, Ye Shugao, Sun Jie, Yang Zhenkun, Chen Jingyu (2019). MicroRNA-221-3p promotes pulmonary artery smooth muscle cells proliferation by targeting AXIN2 during pulmonary arterial hypertension. Vascular Pharmacology.

[3] Lu X, Murphy TC, Nanes MS, Hart CM (2010). PPARγ regulates hypoxia-induced Nox4 expression in human pulmonary artery smooth muscle cells through NF-κB. Am J Phys Lung Cell Mol Phys.

[4] Batista PJ, Chang HY (2013). Long noncoding RNAs: cellular address codes in development and disease. Cell.

[5] Xu X, Ji S, Li W, Yi B, Li H, Zhang H (2017). LncRNA H19 promotes the differentiation of bovine skeletal muscle satellite cells by suppressing Sirt1/FoxO1. Cell Mol Biol Lett.

[6] Rinn JL, Chang HY (2012). Genome regulation by long noncoding RNAs. Annu Rev Biochem.

[7] Du J, Zhang G, Qiu H, Yu H, Yuan W (2019). The novel circular RNA circ-CAMK2A enhances lung adenocarcinoma metastasis by regulating the miR-615-5p/fibronectin 1 pathway. Cell Mol Biol Lett..

[8] Klattenhoff CA, Scheuermann JC, Surface LE, Bradley RK, Fields PA, Steinhauser ML (2013). Braveheart, a long noncoding RNA required for cardiovascular lineage commitment. Cell.

[9] Song X, Shan D, Chen J, Jing Q (2014). miRNAs and lncRNAs in vascular injury and remodeling. Sci China Life Sci.

[10] Wu G, Cai J, Han Y, Chen J, Huang Z-P, Chen C (2014). LincRNA-p21 regulates neointima formation, vascular smooth muscle cell proliferation, apoptosis, and atherosclerosis by enhancing p53 activity. Circulation.

[11] Huang T, Wang J, Zhou Y, Zhao Y, Hang D, Cao Y. LncRNA CASC2 is up-regulated in osteoarthritis and participates in the regulation of IL-17 expression and chondrocyte proliferation and apoptosis. Biosci Rep. 2019;39(5). 10.1042/BSR20182454 PMC6522707 31015370

[12] Xing HB, Qiu HM, Li Y, Dong PF, Zhu XM (2019). Long noncoding RNA CASC2 alleviates the growth, migration and invasion of oral squamous cell carcinoma via downregulating CDK1. Eur Rev Med Pharmacol Sci.

[13] Gao X, Du H, Zhang R, Li C, Wang H, Xuan Q (2019). Overexpression of cancer susceptibility candidate 2 inhibited progression of hepatocellular carcinoma cells. J Cell Physiol.

[14] Wang XW, Zhang W (2019). Long non-coding RNA cancer susceptibility candidate 2 inhibits the cell proliferation, invasion and angiogenesis of cervical cancer through the MAPK pathway. Eur Rev Med Pharmacol Sci.

[15] Gong J, Chen Z, Chen Y, Lv H, Lu H, Yan F (2019). Long non-coding RNA CASC2 suppresses pulmonary artery smooth muscle cell proliferation and phenotypic switch in hypoxia-induced pulmonary hypertension. Respir Res.

[16] Deng L, Bradshaw AC, Baker AH (2016). Role of noncoding RNA in vascular remodelling. Curr Opin Lipidol.

[17] Dai H, Xu LY, Qian Q, Zhu QW, Chen WX. MicroRNA-222 promotes drug resistance to doxorubicin in breast cancer via regulation of miR-222/bim pathway. Biosci Rep. 2019;39(7). 10.1042/BSR20190650 PMC6629945 31273056

[18] Gong L, Zhang W, Yuan Y, Xing X, Li H, Zhao G (2018). miR-222 promotes invasion and migration of ovarian carcinoma by targeting PTEN. Oncol Lett.

[19] Liu X, Cheng Y, Yang J, Xu L, Zhang C (2012). Cell-specific effects of miR-221/222 in vessels: molecular mechanism and therapeutic application. J Mol Cell Cardiol.

[20] Liu X, Cheng Y, Zhang S, Lin Y, Yang J, Zhang C (2009). A necessary role of miR-221 and miR-222 in vascular smooth muscle cell proliferation and neointimal hyperplasia. Circ Res.

[21] Liu XL, Meng J, Zhang XT, Liang XH, Zhang F, Zhao GR (2019). ING5 inhibits lung cancer invasion and epithelial-mesenchymal transition by inhibiting the WNT/beta-catenin pathway. Thorac Cancer.

[22] Zhu TT, Sun RL, Yin YL, Quan JP, Song P, Xu J (2019). Long noncoding RNA UCA1 promotes the proliferation of hypoxic human pulmonary artery smooth muscle cells. Pflugers Arch.

[23] Rabinovitch M (2012). Molecular pathogenesis of pulmonary arterial hypertension. J Clin Invest.

[24] Barrier M, Meloche J, Jacob MH, Courboulin A, Provencher S, Bonnet S (2012). Today’s and tomorrow's imaging and circulating biomarkers for pulmonary arterial hypertension. Cell Mol Life Sci.

[25] Chen D, Gao W, Wang S, Ni B, Gao Y (2017). Critical effects of epigenetic regulation in pulmonary arterial hypertension. Cell Mol Life Sci.

[26] Zhang H, Liu Y, Yan L, Wang S, Zhang M, Ma C (2019). Long noncoding RNA Hoxaas3 contributes to hypoxia-induced pulmonary artery smooth muscle cell proliferation. Cardiovasc Res.

[27] Sun Z, Nie X, Sun S, Dong S, Yuan C, Li Y (2017). Long non-coding RNA MEG3 Downregulation triggers human pulmonary artery smooth muscle cell proliferation and migration via the p53 signaling pathway. Cell Physiol Biochem.

[28] Zhuo Y, Zeng Q, Zhang P, Li G, Xie Q, Cheng Y (2017). Functional polymorphism of lncRNA MALAT1 contributes to pulmonary arterial hypertension susceptibility in Chinese people. Clin Chem Lab Med.

[29] Maegdefessel L, Rayner KJ, Leeper NJ (2015). MicroRNA regulation of vascular smooth muscle function and phenotype: early career committee contribution. Arterioscler Thromb Vasc Biol.

[30] Huang Y (2018). The novel regulatory role of lncRNA-miRNA-mRNA axis in cardiovascular diseases. J Cell Mol Med.

[31] Ding S, Huang H, Xu Y, Zhu H, Zhong C (2017). MiR-222 in Cardiovascular Diseases: Physiology and Pathology. Biomed Res Int.

[32] Xu Y, Bei Y, Shen S, Zhang J, Lu Y, Xiao J (2017). MicroRNA-222 promotes the proliferation of pulmonary arterial smooth muscle cells by targeting P27 and TIMP3. Cell Physiol Biochem.

[33] Zhao QY, Ju F, Wang ZH, Ma XZ, Zhao H (2015). ING5 inhibits epithelial-mesenchymal transition in breast cancer by suppressing PI3K/Akt pathway. Int J Clin Exp Med.

[34] Zhang F, Zhang X, Meng J, Zhao Y, Liu X, Liu Y (2015). ING5 inhibits cancer aggressiveness via preventing EMT and is a potential prognostic biomarker for lung cancer. Oncotarget.

