# Supplementary figures and images for: LncRNA CASC2 inhibits hypoxia-induced pulmonary artery smooth muscle cell proliferation and migration by regulating the miR-222/ING5 axis
Source: Cell Mol Biol Lett. 2020 Mar 17;25:21. doi: 10.1186/s11658-020-00215-y (PMC7079380; doi:10.1186/s11658-020-00215-y)

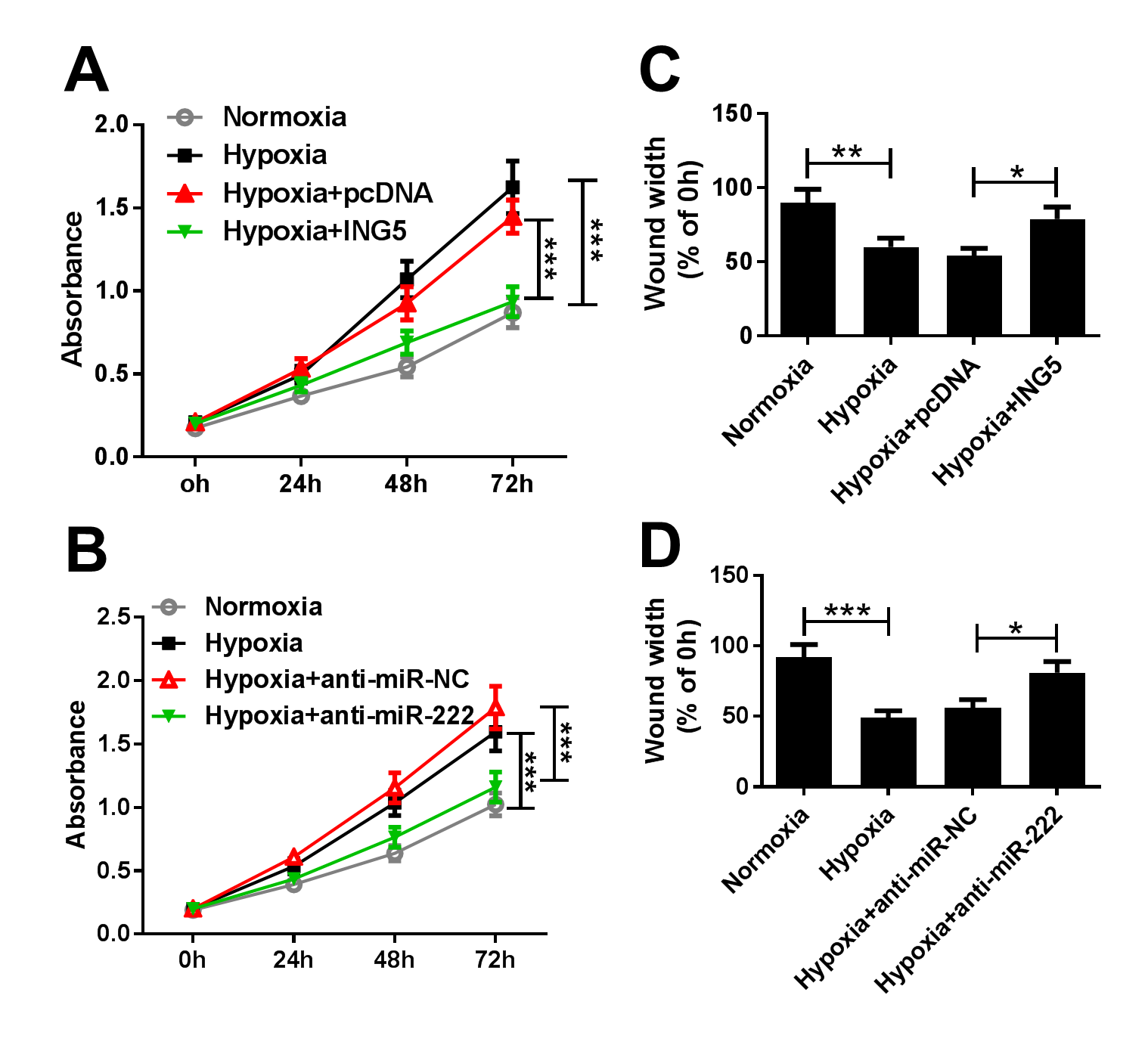

Supplement: Supplementary file 1 — Additional file 1 Figure S1. Effects of miR-222 and ING5 on proliferation and migration of hypoxia-induced PASMCs. (A, C) Effects of miR-222 inhibition on proliferation and migration of normoxia- or hypoxia-induced PASMCs were detected using CCK-8 assay or wound healing assay. (B, D) Effects of ING5 overexpression on proliferation and migration of hypoxia-induced PASMCs were detected using CCK-8 assay or wound healing assay. Experiments were performed three times. *P < 0.05, **P < 0.01, ***P < 0.001. [file 11658_2020_215_MOESM1_ESM.tif]
